# Blockade of TLR2 and TLR4 Attenuates Inflammatory Response and Parasite Load in Cutaneous Leishmaniasis

**DOI:** 10.3389/fimmu.2021.706510

**Published:** 2021-10-06

**Authors:** Pedro Paulo Carneiro, Andreza S. Dórea, Walker N. Oliveira, Luiz Henrique Guimarães, Claúdia Brodskyn, Edgar M. Carvalho, Olívia Bacellar

**Affiliations:** ^1^ Serviço de Imunologia, Hospital Universitário Prof. Edgard Santos, Universidade Federal da Bahia, Salvador, Brazil; ^2^ Faculdade de Medicina, Universidade Federal do Sul da Bahia, Teixeira de Freitas, Brazil; ^3^ Goncalo Moniz Institute (IGM), Fiocruz, Salvador, Brazil; ^4^ Instituto Nacional de Ciência e Tecnologia de Doenças Tropicais - INCT-DT Conselho Nacional de Desenvolvimento Científico e Tecnológico/ Ministério da Ciência e Tecnologia (CNPq/MCT), Salvador, Brazil

**Keywords:** innate immunity, cytokines, cutaneous leishmaniasis, *Leishmania braziliensis*, toll-like receptors

## Abstract

Human cutaneous leishmaniasis (CL) caused by *Leishmania braziliensis* is characterized by a pronounced inflammatory response associated with ulcer development. Monocytes/macrophages, the main cells harboring parasites, are largely responsible for parasite control. Toll-like receptor (TLR) signaling leads to the transcription of inflammatory mediators, such as IL-1β and TNF during innate immune response. TLR antagonists have been used in the treatment of inflammatory disease. The neutralization of these receptors may attenuate an exacerbated inflammatory response. We evaluated the ability of TLR2 and TLR4 antagonists to modulate host immune response in *L. braziliensis*-infected monocytes and cells from CL patient skin lesions. Following TLR2 and TLR4 neutralization, decreased numbers of infected cells and internalized parasites were detected in CL patient monocytes. In addition, reductions in oxidative burst, IL-1β, TNF and CXCL9 production were observed. TNF production by cells from CL lesions also decreased after TLR2 and TLR4 neutralization. The attenuation of host inflammatory response after neutralizing these receptors suggests the potential of TLR antagonists as immunomodulators in association with antimonial therapy in human cutaneous leishmaniasis.

## Introduction

Human cutaneous leishmaniasis (CL) caused by *Leishmania braziliensis* is characterized by an exacerbated cellular immune response and scarce numbers of parasites in lesions (1). While the presence of pro-inflammatory cytokines, such as IFN-*γ* and TNF, are important for the control of parasite proliferation, total pathogen clearance is not achieved. Moreover, an exaggerated Th1 immune response has been associated with severe inflammation and disease pathology. Notably, levels of IL-10, a regulatory cytokine, remain low or absent (2–5).

Following migration from peripheral blood to sites of inflammation, monocytes differentiate into macrophages that play an important role in antigen presentation and *Leishmania* killing (6, 7).

The invasion and survival of *Leishmania* within host cells at early stages of infection involves interactions between molecules present on the surfaces of parasites and monocyte/macrophage receptors (8). Several studies have shown that different receptors mediate the internalization and phagocytosis of *Leishmania* spp. by macrophages (9). Recently, the participation of toll-like receptors (TLR) in protozoan recognition has been highlighted with regard to cytokine production, as well as in the generation of an effective inflammatory response (8). The binding of leishmania molecules to TLRs triggers the release of mediators, including cytokines, which initiate and regulate inflammatory responses necessary for controlling parasite proliferation and orchestrating the development of an adaptive immune response (10, 11).

Many studies investigating the role of TLRs in leishmaniasis have been performed in experimental models, limiting our understanding of the roles played by these receptors in the context of human leishmaniasis. Macrophages from MyD88^-^/^-^TRIF^-^/^-^ *L. panamensis*-infected C57BL/6 mice, which are unable to activate TLR-dependent pathways, present a decreased ability to secrete TNF and increased parasite burden at early times of infection (12). In contrast, the absence of the receptor in TLR2-deficient C57BL/6 mice infected with *L. amazonensis* decreased parasitic load and enhanced the recruitment of inflammatory cells to the site of infection at early infection stages (13). Viana et al. demonstrated higher TLR2 expression in human monocytes infected with *L. braziliensis* compared to *Leishmania infantum*, which was also found to positively correlate with TNF production (14). Our previous work has shown that monocytes from CL patients express *ex vivo* and after infection with *L.braziliensis* more TLR2 and TLR4 than monocytes obtained from healthy individuals; again, this was linked to TNF expression (15, 16).

Although pentavalent antimony is the first line of treatment for CL, high rates of therapeutic failure have been documented (17, 18). Some studies have shown that the administration of antimony in association with immunoregulatory drugs leads to more efficacious treatment outcomes than antimony alone (19–21). Natural and synthetic TLR antagonists, which reduce TLR signaling and effector functions, have been evaluated in preclinical and clinical models of inflammatory disease (22). The blockade of TLR2 by OPN301 (an IgG1 monoclonal anti-TLR2 antibody) in cells of patients with rheumatoid arthritis was found to decrease the production of TNF, IL-1β and IFN-y after stimulation with Pam3Cysk4 (a TLR2 agonist) (23). The present study endeavored to evaluate whether TLR2 and TLR4 neutralization leads to impaired monocyte infection, consequently modulating the inflammatory response observed in American tegumentary leishmaniasis (ATL) caused by *L. braziliensis*.

## Material and Methods

### Patients

A total of 34 CL patients and 10 healthy subjects were included. All patients were examined at the Municipal Health Clinic of Corte de Pedra (Bahia, Brazil), located in a known region of *L*. *braziliensis* transmission. Diagnostic criteria included a clinical presentation characteristic of CL in conjunction with parasite isolation in culture, pathogen identification *via* histopathologic analysis, or *L*. *braziliensis* positivity by PCR. All patients were treated with i.v. pentavalent antimonial (Sb^v^) (meglumine antimoniate; Sanofi-Aventis, Paris-France) daily at 20 mg/kg of body weight for 20 days. All experiments were performed prior to the administration of therapy. The CL group consisted of 20 males and 14 females with a median age of 45 years (range: 18-46). The control group was formed by 10 healthy subjects (HS) living in an urban area without exposure to *L*. *braziliensis*: 5 males and 5 females, median age 30 years (range: 22-32). The present study received approval from the Institutional Review Board of the Professor Edgard Santos University Hospital Complex (HUPES-UFBA, protocol no. 693.111). Terms of informed consent were obtained from all study participants.

### Parasite Cultures

*L*. *braziliensis* (MHOM/BR/2003/LTCP11245) isolates obtained from a skin lesion of a CL patient were initially cultivated in biphasic medium (NNN). Following isolation, parasites were cryopreserved in frozen nitrogen. None of the parasites selected for this study had been previously cultivated in liquid culture medium. After selection, parasites were expanded in Schneider’s complete medium and identified as *L*. *braziliensis* by multilocus enzyme electrophoresis (24). All reagents and Schneider’s medium were determined to be endotoxin-free by limulus amebocyte lysate (LAL) bacterial endotoxin testing (BioReliance, SIGMA-ALDRICH). We used *L. braziliensi*s promastigotes in the stationary phase (14).

### Soluble *Leishmania* Antigen Preparation

Soluble *Leishmania* antigen (SLA) was prepared from *L. braziliensis* (MHOM/BR/2001) isolated from a patient with CL as previously described (25). This antigen was tested for endotoxins by LAL and used at a concentration of 5 μg/mL.

### Evaluation of TLR2 and TLR4 Expression by Flow Cytometry

Peripheral blood mononuclear cells (PBMC) were isolated from heparinized venous blood by Ficoll-Hypaque density gradient centrifugation (GE Healthcare), washed and suspended in RPMI 1640 medium (supplemented with 5% fetal calf serum, 100U penicillin/mL, 100μg streptomycin/mL) (GIBCO BRL, Grand Island, NY, USA).

CD14, TLR2 and TLR4 expression were analyzed in PBMC from CL patients and healthy controls infected with *L. braziliensi*s promastigotes in the stationary phase at 5:1monocyte, considering that 10% of PBMC are monocytes, for 2 hours. As positive controls for TLR expression, PBMC were stimulated for 2 hours with 100ng/mL of either LPS (Lipopolysaccharide) or Pam3Cys (Pam3cys-Ser- (Lys) 4) (37°C, 5% CO_2_). Anti-CD14 (APC, BD bioscience, Clone M5E2), anti-TLR2 PE (CD282, clone TL2.1, Invitrogen), anti-TLR4 PE (CD284,clone HTA125, Invitrogen) and anti-TLR4 FITC (clone HTA125, IMGENEX) were used for the labeling of surface molecules. Receptor expression was analyzed by flow cytometry, with 200,000 events acquired per sample. Data analysis was performed using FlowJo software (Free Star Inc.).

### Evaluation of *L. braziliensis* Infection and Parasite Viability Following TLR2 and TLR4 Neutralization

For the neutralization of TLR2 and TLR4, PBMC were incubated for 1 hour (37°C, 5% CO_2_) in polystyrene tubes containing RPMI 1640 medium with 100uM each of anti-TLR2 and anti-TLR4 (Abcam). Following TLR2 and TLR4 neutralization, PBMC were infected with *L. braziliensis* stationary phase promastigotes (5:1) for 2 or 48 hours (37°C, 5% CO_2_). After infection, the numbers of infected cells and intracellular parasites were determined through the microscopic evaluation of 100 monocytes, employing May Grunewald-Giemsa staining from cytocentrifuge preparations. Alternatively, after 72 hours of infection, monocytes were washed and RPMI medium was replaced with 0.5 mL of Schneider’s medium (Sigma-Aldrich) supplemented with 10% fetal calf serum to quantify the number of viable parasites. Cells were then cultured for an additional 72 hours at 26°C. Finally, viable extracellular motile promastigotes were counted on a hemocytometer (26, 27). Also, PBMC were infected at a ratio of 5:1 for 2 hours with fluorescent GFP-*L.braziliensis* (MHOM/BR/00/BA/866) grown in Schneider’s culture medium supplemented with 20% fetal bovine serum, 100 U/mL penicillin and 100 μg/ml streptomycin, and then maintained at 24°C.

### Evaluation of Oxidative Burst

Following the neutralization of TLR2 and TLR4, PBMC-derived monocytes (1x10^6^) were stimulated with 10 ng/mL dihydrorhodamine 123 (DHR) (Cayman Chemical Company) for 10 minutes (37 °C, 5% CO_2_). Cells were then exposed to *L. braziliensis* stationary phase promastigotes (ratio 5:1) for 25 minutes (37°C, 5% CO_2_). Phorbol 12-myristate 13-acetate (PMA-Invivogen) at 1µg/mL was used as a positive control. Monocytes were stained for anti-CD14 surface markers (APC clone M5E2, BD Pharmingen) and quantified by nonspecific fluorescence using forward scatter (FSC) and side scatter (SSC) parameters to determine cell size and granularity, respectively. Cells were then gated based on CD14 expression and DHR oxidation (**Figure 4A**). A total of 200,000 events were evaluated on a FACS Canto™ II flow cytometer (BD). Data analysis was performed using FlowJo software (Tree Star Inc).

### Cytokine and Chemokine Production Following TLR2 and TLR4 Neutralization

After neutralizing TLR2 and TLR4 in PBMC as described above, cells were infected with *L. braziliensis* (5:1) and cultured for 48 hours (37°C, 5% CO_2_). Cytokine and chemokine (IL-1β, TNF-α, IL-10, CXCL9 and CXCL10) production were measured after 48 hours in supernatants using the sandwich ELISA technique (BD Bioscience Pharmingen, San Jose, CA, USA).

For the evaluation of intracellular cytokine and chemokine production, PBMCs were infected or not with *L. braziliensis* stationary phase promastigotes at 5:1, for 48h (37°C, 5% CO_2_). Cells were then stained with anti-CD14 monoclonal antibodies (APC), washed with PBS (1,500 rpm, 5 min, 4°C), fixed with 4% paraformaldehyde, and permeabilized with BD Perm/Wash™ solution (BD-Bioscience) for 15 min at 4°C in the absence of light. Intracellular staining was performed with anti-TNF/PerCP Cy-5.5 (BD Pharmingen™, Clone MAb11), anti-IL-10/FITC(eBioscience, Clone JES5-16E3), anti-IL1β/PE-Cy7(eBioscience, Clone NJTEN3), anti-CXCL9/FITC(eBioscience, Clone 49106) and anti-CXCL10/PE(Biolegend, Clone J034D6) antibodies for 30min. Cells were then washed and suspended in 400 µl PBS for flow cytometry analysis on a BD FACS CANTO™ II. A total of 200,000 events were acquired. Data analysis was performed using FlowJo software (Free Star Inc.).

### TLR2, TLR4 and TNF Expression in Monocytes Following SLA Stimulation

PBMCs were isolated and cultured for 2 hours (37°C, 5% CO_2_) in the presence or absence of SLA (5 μg/mL). To evaluate the expression of TLR2 and TLR4, cells were labeled with anti-CD14 (APC) (eBioscience, San Diego, CA, USA), anti-TLR2 PE (CD282, clone TL2.1, invitrogen) and anti-TLR4 PE (CD284,clone HTA125, invitrogen). Receptor expression was assessed by flow cytometry (BD FACS CANTO™ II). A total of 200,000 events were acquired. Data analysis was performed using the FlowJo software (Free Star Inc.).

To evaluate TNF expression, the neutralization of TLR2 and TLR4 was performed as described above and cells were then stimulated with SLA (5 µg/mL) for 6 hours (37°C, 5% CO_2_). All cells were washed with PBS (1,500 rpm, 5 min, 4°C), fixed in 4% paraformaldehyde and permeabilized with BD Perm/Wash™ solution (BD Biosciences) for 15 min at 4°C in the absence of light. Intracellular staining was performed with anti-TNF (PerCP Cy-5.5) antibodies for 30 min, cells were washed and resuspended in 400 µl PBS for flow cytometry analysis on a BD FACS CANTO™ II. A total of 200,000 events were acquired. Data analysis was performed using FlowJo software (Free Star Inc.).

### Evaluation of TLR2 and TLR4 Expression in Cells From CL Patient Biopsies

Skin samples were obtained from CL patient lesions using a 4-mm punch. Biopsies were treated with collagenase for 90 mins (37°C, 5% CO_2_), dissociated and cell suspensions were filtered using a 50 μm Medicon filter (BD pharmingen). To evaluate TLR2 and TLR4 expression, surface molecules were labeled using anti-CD14 (APC) (eBioscience, San Diedo, CA, USA), anti-TLR2 PE (clone TL2.1) and anti-TLR4 PE (clone HTA125) (IMGENEX, San Diego, CA, USA). Receptor expression was analyzed by flow cytometry, with 200,000 events acquired per sample.

In addition, biopsies from *L. braziliensis* patients were cultured in complete RPMI medium in the presence or absence of 100uM anti-TLR2 and 100uM anti-TLR4 for 72 hours (37°C, 5% CO_2_). Biopsy culture supernatants were collected and stored at −70°C until the time of IL-10, IL-1β, TNF and CXCL9 quantification by ELISA (R&D Systems) in accordance with the manufacturer’s instructions. Expression results are expressed in pg/ml.

### Statistical Analysis

The Mann-Whitney test was employed for comparisons, while nonparametric Wilcoxon signed-rank testing was used to compare results obtained from cells derived from a single patient under different experimental conditions. All data are presented as medians and respective interquartile range (IQR). Statistical analyses were performed using GraphPad Prism 4.0 (GraphPad Software, Inc., San Diego, CA, USA), with an alpha value of P<0.05 considered statistically significant.

## Results

### TLR2 and TLR4 Expression Increase in Monocytes From CL Patients During *L. braziliensis* Infection

**Figure 1:**
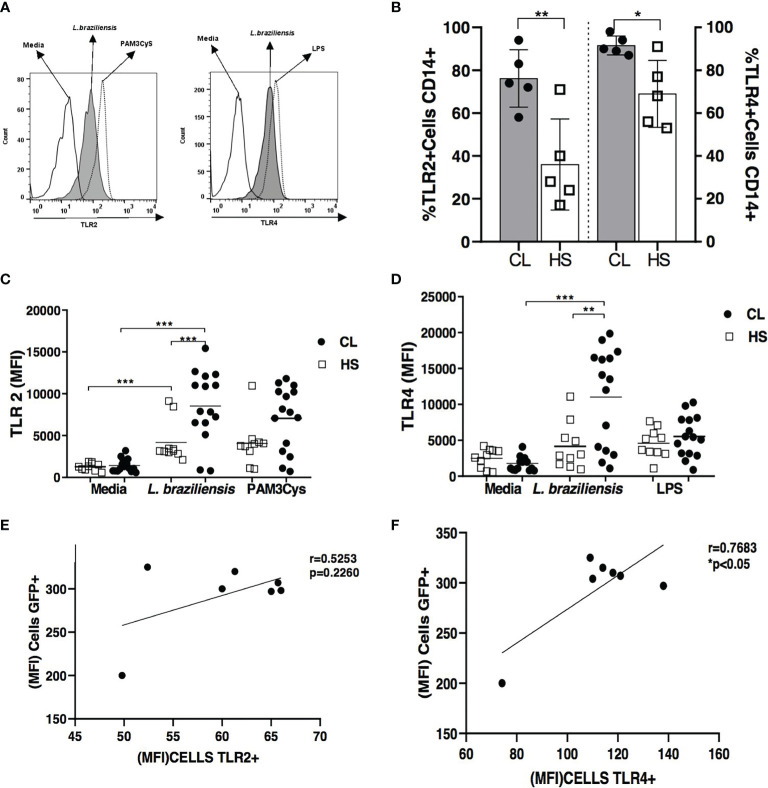
TLR2 and TLR4 expression in *L. braziliensis*-infected monocytes from cutaneous leishmaniasis patients and healthy subjects. Monocytes in PBMC from CL patients (n = 15) and HS (n = 10) were infected with *L. braziliensis* (5: 1) for 2 hours and then the cells were labeled with anti-CD14 antibodies for the characterization of monocytes. Figure **(A)** shows the representative histogram from TLRS expression in CL patients. Frequency of TLR2 and TLR4 receptors in in the same CD14+ cells is showed in Figure **(B)** MFI of TLR2 and TLR4 in CD14^+^ infected cells are presented in figure **(C)** and **(D)** As a positive control of infection, Pam3Cys (TLR2 agonist) and LPS (TLR4 agonist) were used. Receptor expression was performed by flow cytometry. All p values were obtained using the Mann Whitney and Wilcoxon signed-rank test *p < 0.05, **p < 0.01 and ***p < 0.001. The Pearson correlation analyses was performed between GFP MFI and TLR2 **(E)** and TLR4 **(F)**.

The expression of TLR2 and TLR4 receptors was evaluated in monocytes from CL patients and cells from HS after *in vitro* infection with *L. braziliensis* promastigotes (**Figure 1**). First, we observed the frequency of TLR2 and TLR4 in the same cells from CL patients after *L.braziliensis* infection, and the expression of TLR4 was higher than TLR2 74(58-94) *versus* 90(87-98), p<0.05. The same was observed in HS cells, (**Figure 1B**). Median TLR2 and TLR4 expression, represented by mean fluorescence intensity (MFI), was found to be higher in *L. braziliensis-*infected monocytes from CL patients compared to cells from HS (**Figures 1C, D**). We observed a positive correlation between GFP MFI with TLR2 and TLR4. However, only TLR4 MFI has significant correlation with GFP MFI (r= 0.7683 and p<0.05) (**Figures 1E, F**).

### TLR2 and TLR4 Neutralization Decreases the Internalization of *L. braziliensis* in Monocytes From CL Patients

**Figure 2:**
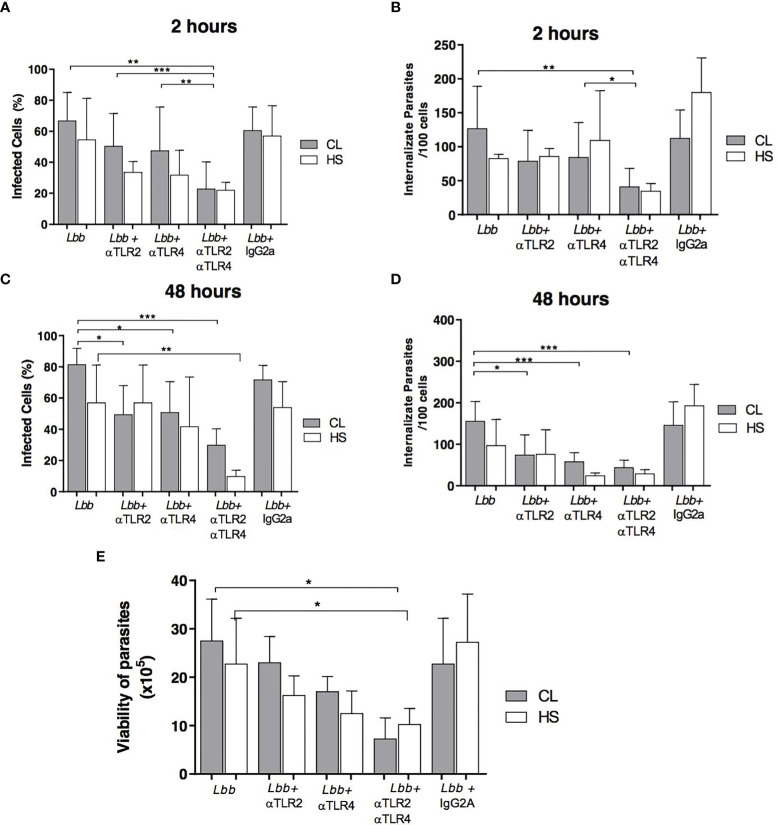
Proportion of infected cells and parasite load after TLR2 and TLR4 neutralization. Monocytes from PBMCs of CL patients (N=9) and HS (N=4) were infected with *L. braziliensis* (5:1) for 2 or 48 hours. Numbers of infected cells after 2 hours **(A)** and 48 hours **(C)**, as well as the number of internalized parasites after 2 hours **(B)** and 48 hours **(D)** were quantified by optical microscopy. Data are representative of median values of infected cells and internalized parasites After 72 hours, RPMI medium was replaced with Schneider’s medium, and the number of viable promastigotes was estimated after 3 days **(E)**. Data are representative of median numbers of viable parasites. All p values were obtained using the Wilcoxon signed-rank test, *p<0.05, **p<0.01, ***p<0.001.

The frequency of *L. braziliensis*-infected cells and numbers of intracellular parasites were quantified in TLR2- and/or TLR4-neutralized monocytes from CL patients and HS at 2 and 48 hours post-infection (**Figure 2**). At 2 hours of infection, a significantly lower frequency of infected cells was observed following the neutralization of both receptors: 21% (11-29) *versus* 73% (54-78) for non-neutralized monocytes (p<0.01) (**Figure 2A**). After 48 hours of infection, a decreased frequency of infected cells was observed following the neutralization of TLR2, 54% (31-62%), and TLR4, 45% (39-62%), *versus* 84% (72-91%) in non-neutralized monocytes (p<0.05) (**Figure 2C**). However, a more pronounced decrease in the number of infected cells occurred following simultaneous TLR2 and TLR4 neutralization: 29% (19-38%) *versus* 84% (72-91%) (p<0.001) (**Figure 2C**). With respect to numbers of intracellular parasites, at 2 hours post-infection the simultaneous neutralization of both receptors provoked a decrease in the number of intracellular parasites: 44 (18-66) *versus* 120 (87-127) for non-neutralized monocytes (p<0.01) (**Figure 2B**). After 48 hours of infection, the neutralization of TLR2 and TLR4 further lowered the number of intracellular parasites, especially when both receptors were neutralized: 43 (30-55) *versus* 138 (121-196) (p <0.001) (**Figure 2D**). There was no difference between CL patients and HS. Another group of monocytes from CL patients was also infected with *L. braziliensis-*GFP for 2 hours following TLR2 and TLR4 neutralization. The frequency of *L. braziliensis*-internalized cells expressing GFP decreased to 10% (3-15%) *versus* 51% (27-62%) in non-neutralized monocytes (p<0.01) (**Supplementary Figure 1**).

### *Leishmania* Viability After TLR2 and TLR4 Neutralization

The viability of *Leishmania* promastigotes was estimated by assessing the proliferation of extracellular motile parasites in infected monocytes from CL patients cultivated in the presence or absence of anti-TLR2 and anti-TLR4 antibodies. We observed that the neutralization of both TLR2 and TLR4 resulted in fewer viable parasites: 8 x10^5^ (2-10x10^5^) in comparison to cells cultured in the absence of antibodies: 26x10^5^ (19-36x10^5^) (p<0.05) (**Figure 2E**).

### TLR2 and TLR4 Neutralization Decreases Oxidative Burst in Monocytes From CL Patients

It has been demonstrated that higher oxidative burst occurs in CL monocytes infected with *L. braziliensis* compared to HS monocytes; rather than controlling parasite growth, this phenomenon has been associated with pathology (15).

**Figure 3:**
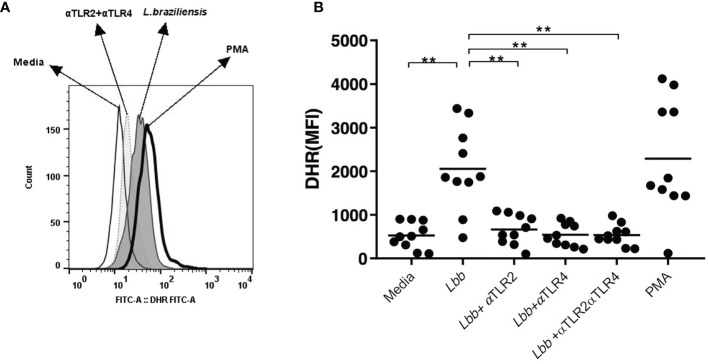
Neutralization of TLR2 and TLR4 interferes with oxidative burst in CL monocytes. Peripheral blood monocytes from CL patients (n = 10) were treated with anti-TLR2 and anti-TLR4 antibodies, infected with *L. braziliensis* (5:1) for 25 minutes. Cells were labeled with anti-CD14 antibody for monocyte characterization. Respiratory burst was measured *via* DHR123 oxidation by flow cytometry. PMA was used as a positive control **(A)**. Data plots represent median mean fluorescence intensity (MFI) values **(B)**. Wilcoxon signed-rank test; **p<0.01.

We evaluated the effects of anti-TLR2 and TLR4 antibodies on oxidative burst production by *L. braziliensis*-infected CL monocytes (**Figure 3A**). In the presence of neutralizing antibodies, a significant decrease in the MFI of DHR was seen in cells from CL patients: 626 (104-1091) for the blockade of TLR2 and 496 (214-922) for TLR4, *versus* 1373 (478-1876) (p<0.01). The neutralization of both receptors further decreased the production of oxidative radicals in monocytes from CL patients to 484 (221-981) *versus* 1373 (478-1876) (p <0.001) (**Figure 3B**). In cells from HS, the neutralization of TLR2 and TLR4 also reduced DHR expression, yet in the absence of statistical significance (data not shown).

### TLR2 and TLR4 Neutralization Decreases TNF and IL-1β Production in Monocytes From CL Patients

**Figure 4:**
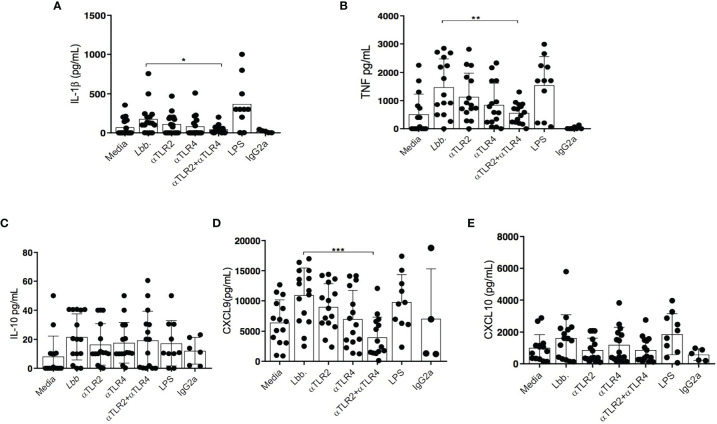
Evaluation of cytokine production by CL monocytes after TLR2 and TLR4 neutralization. Monocytes from CL patients (n = 15) were treated or not with anti-TLR2 and anti-TLR4 antibodies, then infected with *L. braziliensis* (5:1) for 48 hours. IL-1β **(A)** TNF **(B)**, IL-10 **(C)**, CXCL9 **(D)** and CXCL10 **(E)** were quantified by ELISA. Data are representative of median cytokine production. Wilcoxon signed-rank test; *p<0.05, ** p<0.01, ***p<0.001.

An association between IFN-γ and TNF production and lesion size has been demonstrated in *L. braziliensis* infection, and these proinflammatory cytokines have also been linked to more severe forms of disease (4, 5, 28). Furthermore, both inflammasome activation and IL-1β have been associated with disease severity in leishmaniasis (29). We observed lower levels of IL-1β production in *L. braziliensis*-infected monocytes following the simultaneous neutralization of TLR2 and TLR4: 34 pg/ml (4-55) *versus* 125 pg/ml (26-228) in untreated cultures (p<0.05) (**Figure 4A**). TNF production was also observed to decrease significantly in response to simultaneous TLR2 and TLR4 receptor neutralization: 581 pg/ml (229-858) *versus* 1204 pg/ml (496-2527) (p<0.01) (**Figure 4B**). However, the neutralization of these receptors did not alter IL-10 production (**Figure 4C**).

### TLR2 and TLR4 Neutralization Decreases the Production of CXCL9 in Monocytes From CL Patients

A previous study documented that *L. braziliensis*-infected macrophages from CL patients produce more CXCL9 than macrophages obtained from individuals with subclinical infection or cells from HS (30). Accordingly, we investigated the effect of TLR neutralization on the production of CXCL9 and CXCL10, chemokines triggered by IFN-γ. The production of CXCL9 was observed to decrease significantly after simultaneously neutralizing both receptors: 2,420 pg/ml (120-12,066) *versus* 11,693 pg/ml (2,522-17,000) (p<0.001) (**Figure 4D**). In contrast, no differences in CXCL10 levels were observed in the presence of neutralizing antibodies (**Figure 4E**).

### Evaluation of TLR2 and TLR4 Expression After Stimulation With SLA

As the neutralization of TLR2 and TLR4 was observed to decrease the internalization and numbers of *L. braziliensis-*infected monocytes, cells were stimulated with SLA to investigate the modulation of inflammatory molecule production.

**Figure 5:**
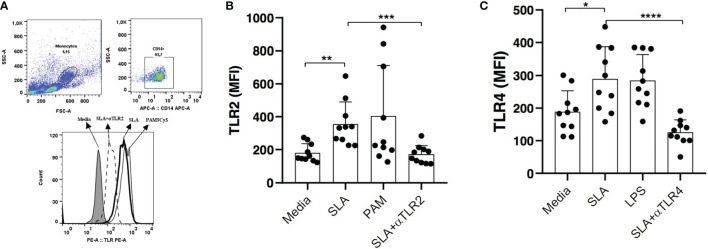
Expression of TLR2 and TLR4 after stimulation with leishmania soluble antigen (SLA). PBMCs from CL patients (n=10) were stimulated with 5ug/ml SLA for 2 hours. Pam3Cys (a TLR2 agonist) and LPS (a TLR4 agonist) were used as positive controls **(A)**.The expression of TLR2 **(B)** and TLR4 **(C)** were quantified by flow cytometry. Data are representative of median mean fluorescence intensity (MFI) values. Wilcoxon signed-rank test; *p<0.05, **p<0.01, ***p<0.001.

Stimulation with SLA was found to upregulate TLR2 expression, as determined by MFI in monocytes from CL patients: 423 (225-648) *versus* 152 (122-274) in unstimulated cells *(*p<0.01) (**Figures 5A, B**). In addition, higher TLR4 expression was also noted: 290 (158-385) *versus* 188 (112-301) (p<0.05) (**Figure 5C**).

**Figure 6:**
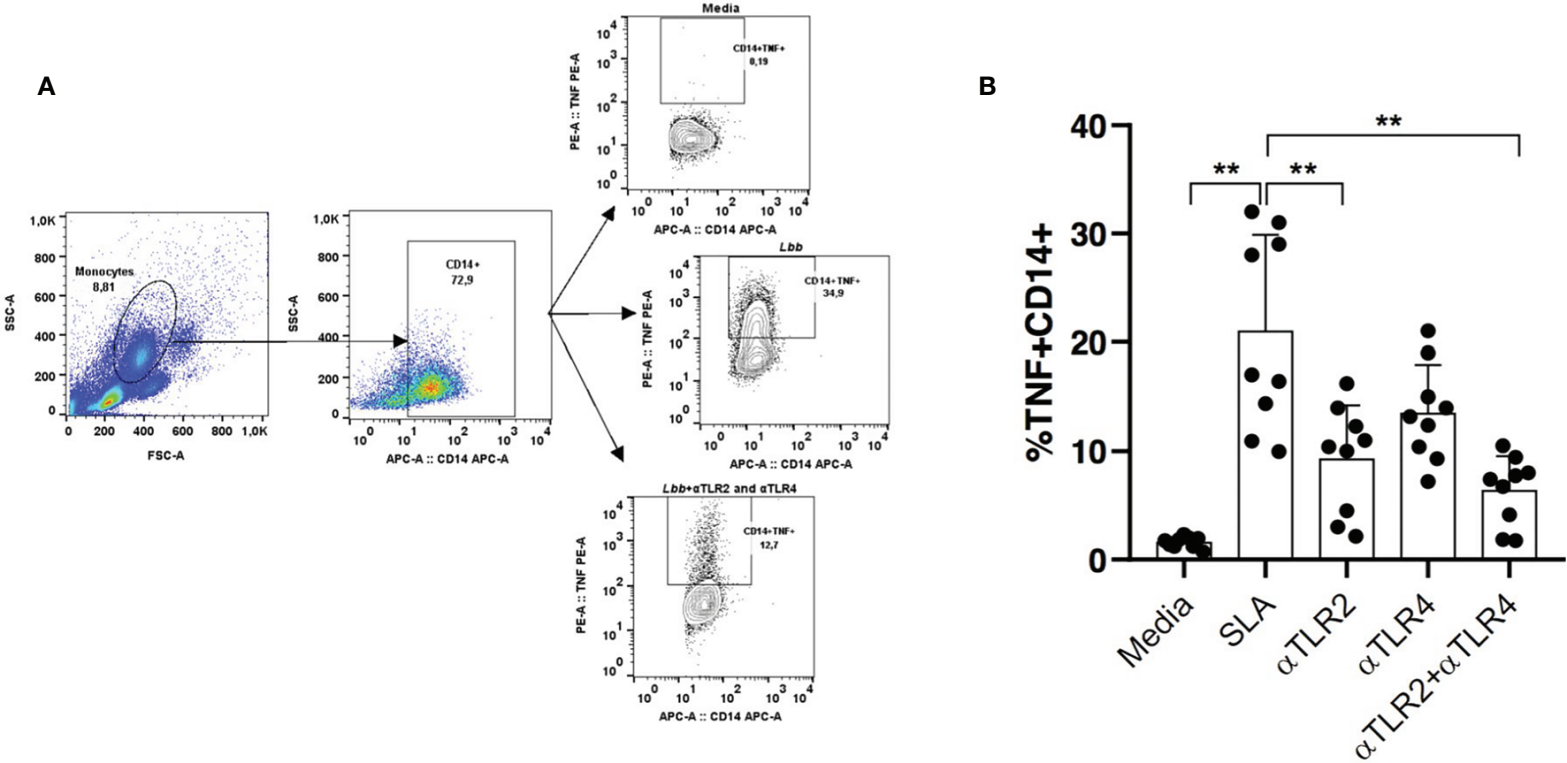
The TNF production after stimulation with leishmania soluble antigen (SLA) and TLR2 and TLR4 neutralization. Monocytes from CL patients (n = 10) were treated or not with anti-TLR2 and anti-TLR4 antibodies. Cells were stimulated with SLA for 8 hours. **(A)** The positive florescence in the population cells were obtained by the strategy using FMO (Florescence Minus One). The frequency of cells expressing TNF in CD14+ cells **(B)** was performed by the flow cytometry technique. All p values were obtained using Wilcoxon’s statistical test **p<0.01.

We further evaluated the impact of simultaneous TLR2 and TLR4 neutralization on TNF production in CD14^+^ cells. Significantly lower levels of TNF were observed after receptor neutralization: 7.4% (1.74%-10.5%) *versus* 17% (9.9%-32%) (**Figures 6A, B**).

### Effect of TLR2 and TLR4 Neutralization on Inflammatory Molecule Production in Cells From CL Skin Biopsies

The inflammatory infiltrate in CL lesions is known to be characterized by cells that produce inflammatory cytokines, such as TNF, IFN-γ and IL1β (4, 31). A recent study evaluating cells from CL patient skin biopsies reported increased IL-1β production in the supernatants of cultured cells compared to healthy skin biopsies (31). Based on these findings, we investigated whether the neutralization of TLR2 and TLR4 would interfere with the production of proinflammatory molecules (IL-1β, TNF, CXCL9) and IL-10 in biopsy cultures from CL patients.

**Figure 7:**
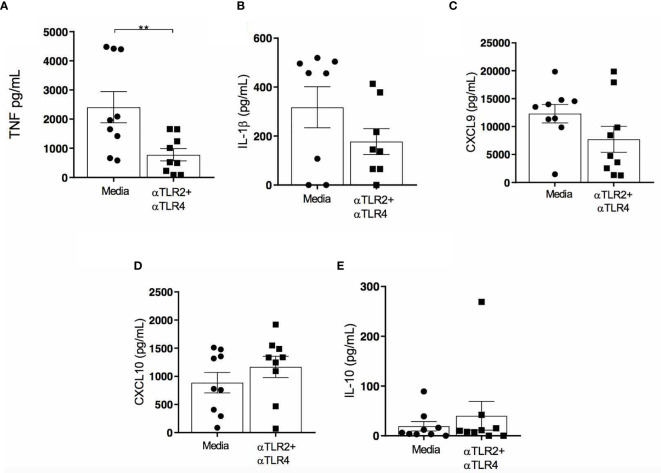
Effects of TLR2 and TLR4 neutralization on cytokine/chemokine production in biopsied cells from CL patient lesions. Cells from the lesions of patients with CL (n=10) were obtained and cultured for 48h in the presence or absence of anti-TLR2 and anti-TLR4. TNF **(A)**, IL-1β **(B)**, CXCL9 **(C)** CXCL10 **(D)** and IL-10 **(E)** were measured in culture supernatants by ELISA. Data are representative of median cytokine/chemokine levels. All p values were obtained using Wilcoxon signed-rank test; **p<0.01.

In CD14^+^ cells obtained from CL patient biopsies, we observed lower TLR2 expression compared to TLR4: 100 (91 - 107) *versus* 152 (131-161), respectively (p<0.01). After neutralizing TLR2 and TLR4, TNF production decreased to 517 pg/ml (84-1662 pg/ml) from 1965 pg/ml (581-4480 pg/ml) (p<0.01) the in supernatant of CL biopsy cell cultures (**Figure 7A**). In addition, the neutralization of both receptors decreased IL-1β production: 141 pg/ml (65-413) *versus* 456 pg/ml (26-519 pg/ml) (p<0.05), **Figure 7B**. CXCL9 production was also observed to decrease following TLR2 and TLR4 neutralization, yet without statistical significance (**Figure 7C**). No differences were detected in CXCL10 (**Figure 7D**) and IL-10 production (**Figure 7E**) as a result of TLR neutralization.

## Discussion

Evidence has shown that an exacerbated inflammatory response is associated with the development of ulcers in cutaneous leishmaniasis caused by *L. braziliensis*. It follows that the identification of mechanisms capable of modulating this response may aid in the development of immunomodulatory therapies. The premise of our study was based on previous reports demonstrating that monocytes from CL patients express more TLR2 and TLR4 in association with elevated TNF production (16, 32). The present results indicate that the neutralization of these receptors can indeed modulate the exacerbated inflammatory response documented in CL caused by *L. braziliensis*.

At early times of infection, the ability of *Leishmania* to invade and survive within the host is dependent on interactions between molecules present in parasites and host cell receptors, which also influence the outcome of infection. Toll-like receptors, especially TLR2 and TLR4, have been implicated in the recognition of various *Leishmania* species (8). Different of human CL, it is well known that during active human visceral leishmaniasis, cell-mediated immune responses are suppressed and consequently a decrease in IFN-γ which is related to the production of regulatory cytokines such as IL-10. In these patients, increased TLR2 and TLR4 expression in lymphocytes and monocytes was associated with increased production of TNF-α, IL-10 and TGF-β and decreased production of IFN-γ and NO (33). In diffuse cutaneous (DCL) leishmaniasis that is characterized by uncontrolled parasite dissemination and poor production of IFN-γ patients showed reduced NK cell numbers that down-regulated TLR2, TLR1 and TLR6 expression as well as reduced cytokine production, as compared to CL patients (34).

The *in vitro* neutralization of TLR2 and TLR4 led to decreased parasitic load and fewer infected monocytes derived from CL patients. Several studies have previously demonstrated low parasitic load and resistance to infection in TLR2- or TLR4-deficient experimental animal models (13, 35).

Our findings indicate that both receptors are important for parasite internalization, since neutralization decreased the frequency of infected cells and numbers of intracellular parasites after two hours of infection. These results were then confirmed in experiments employing GFP-*L. braziliensis* to infect monocytes from CL patients, as flow cytometry revealed a significant decrease in the frequency of infected cells after the simultaneous blockade of both receptors (**Supplementary Figure 1**).

In *Leishmania* infection, neutrophils, the first cell type to migrate to the site of infection, are responsible for parasite death (36). A study involving healthy *L. amazonensis-*infected human neutrophils revealed the expression of TLR2 and TLR4 by these cells. Receptor neutralization subsequently decreased the numbers of infected cells and intracellular parasites, suggesting that these receptors may participate in the internalization of leishmania by other innate immune cells (37).

Parasite proliferation inside phagocytic cells leads to cell lysis and the release of *Leishmania* (26). Herein, similarly to a reduction in the number of intracellular parasites, we also noted a decrease in the release of viable *L. braziliensis* promastigotes following the blockade of TLR2 and TLR4, which reinforces the notion that these receptors participate in the internalization of *L. braziliensis* by host monocytes. There are several reports in literature demonstrating an important role of TLR2 and TLR4 in the infection by *Leishmania*. However, these papers have not clarified the exact role of these receptors in the uptake of parasites, existing controversial depending on the cell type as well as the *Leishmania* species (37). Our data support the idea that both receptors seem to be important in the uptake of *L.braziliensis*, since the TLR2 and TLR4 blockade is the only condition able to decrease the number of internalized leishmania. Alternatively, these receptors may be included in a lipid raft that contains different phagocytic receptors already described for *Leishmania* (38, 39).

Our group previously demonstrated that monocytes from CL patients infected with *L. braziliensis* presented greater respiratory burst compared to cells from HS, which was observed to decrease after treatment leading to lesion cure (15). Here we found that the blockade of TLR2 and TLR4 decreased respiratory burst in infected CL monocytes. Contact between *Leishmania* lipophosphoglycans (LPG) and TLR4 stimulates the synthesis of NADPH oxidase, thus increasing the production of reactive oxygen species. However, the mechanisms underlying TLR-mediated stimulation of oxidative radical production have not been well elucidated. Srivastava et al. (40) demonstrated elevated TLR2 expression in mouse macrophages infected with *L. major*, which was associated with a higher oxidative response induced by the recognition of LPG by TLR2. Consequently, these authors noted the activation of MyD88 and increased iNOS expression (40).

Although the production of these oxidative radicals has been linked to parasite elimination in mice, the role of NO in *L. braziliensis* killing in humans remains unclear. In fact, in CL caused by this species of leishmania, NO production has been correlated with lesion size (15). Hence, it follows that the blockade of TLRs may be an important tool for the inhibition of oxidative radical production, which is more strongly associated with disease pathogenesis than parasite elimination. Although the decline in ROS levels could lead to an increase in the number of parasites, our hypothesis is that the block of TLR2 and 4 lead to a decrease in the uptake of *Leishmania*, decreasing the parasite burden and therefore, allowing a better response to control parasites and possibly *in vivo*, decreasing the inflammatory responses.

Several studies have previously demonstrated that the activation of TLR2 and TLR4, as well as the subsequent activation of transcription factors leading to the production of inflammatory molecules, are important in the control of infection by various *Leishmania* spp. (41, 42). Among these molecules, TNF, which is also involved in the control of infection, plays an important role in tissue damage in the context of human disease caused by *L. braziliensis*. Thus, the present study investigated whether the blockade of TLR2 and TLR4 could interfere with the production of inflammatory molecules by CL patient monocytes. Our evaluation of the production of TNF and IL-1β revealed markedly reduced TNF production following the neutralization of both receptors. LPG, a molecule present on the surface of leishmania that stimulates the production of IL-12 and TNF through the activation of MyD88, constitutes an important ligand for TLR2 (43). Other studies have also shown that LPG and glycoinositol phospholipids, another type of molecule present on the surface of *L. braziliensis*, induce the production of TNF and NO through binding with TLR4 (44–46). Thus, since TLR2 and TLR4 are clearly involved in the production of TNF, the neutralization of these receptors expectedly led to decreased production of this cytokine.

IL-1β, a cytokine associated with various inflammatory diseases (31, 47), has also been associated with the pathogenesis of cutaneous leishmaniasis (48–50). As the signaling of TLRs *via* MyD88, resulting in the activation of NF-ĸβ, can also lead to the transcription of inflammatory gene components, mainly IL-1β (51, 52), we further evaluated whether TLR blocking could modulate the production of this cytokine. Similar to what was observed with TNF, the neutralization of both receptors decreased the production of IL-1β. Our results are agreed with studies that demonstrate the synergy between the TLR2 and TLR4 for the induction of the immune response (53, 54).

Cells from patients with CL caused by *L. braziliensis* produce either low levels of IL-10 or none at all. Although present in the lesions of these patients, this cytokine’s regulatory action may be impaired by the absence of its receptors (3, 4). A study conducted in CL patients with mucosal leishmaniasis demonstrated that the neutralization of IFN-γ decreased TNF production in association with increased levels of IL-10, suggesting that the excessive production of IFN-γ and TNF is associated with the absence or attenuation of a strong IL-10-mediated inflammatory response (28). Thus, we evaluated whether decreases in TNF could also affect the production of IL-10 by CL monocytes. We found no changes in the levels of this cytokine following TRL receptor blocking, which suggests that different pathways are involved in the production of TNF and IL-10 in human CL caused by *L. braziliensis*. Our results are divergent from those reported by Galdino et al. (55), who demonstrated that the blockade of TLR4 decreased IL-10 production (55). Importantly, in contrast to our experimental model, these authors used PBMCs from healthy individuals primed with IFN-γ and subsequently infected them with amastigote forms of *L. braziliensis*, which could help explain this discrepancy. Alternatively, it has also been reported that CD4^+^ CD25^−^ FOXP3^−^ cells are the source of IL-10 production in CL patients (56).

In addition to the production of proinflammatory cytokines, *Leishmania* infection induces the expression of numerous genes related to the production of chemokines (35, 57). Our results indicate that the expression of TLR2 and TLR4 is associated with CXCL9 production, but not CXCL10.

CXCL9 and CXCL10, chemokines important to the recruitment and activation of Th1-type cells, are notable for participation in the pathogenesis of several inflammatory diseases (58, 59). The activation of TLR2 and TLR4 can also activate the transcription of CXCL9 and CXCL10 *via* the phosphorylation of IRF3, an interferon regulatory factor (60). In human CL caused by *L. braziliensis*, macrophages from CL patients produce high levels of CXCL9 compared to cells obtained from individuals with subclinical infection or healthy subjects (30). High systemic production of CXCL9 has been noted in CL caused by *L. braziliensis*, which becomes even higher in disseminated forms of disease, constituting an emerging and more severe form of CL (61). Another study found the production of this chemokine by PBMCs from CL patients stimulated with SLA to be associated with therapeutic failure to pentavalent antimony (62). Thus, CXCL9 seems to contribute more to the pronounced inflammatory response observed in CL than CXCL10. While no studies in the literature have linked the expression of TLRs to chemokine production in *Leishmania* infection, some reports on inflammatory disease did detect an association. In autoimmune arthritis, for instance, the stimulation of TLR2 and TLR4 using agonists resulted in a substantial increase in CXCL9 production, which supports the role of these receptors in the expression of this chemokine (63, 64).

Since a diminished inflammatory immune response could also occur due to weaker infection that consequently affects the proliferation of parasites in monocytes, we also evaluated the effects of TLR-neutralizing antibodies following stimulation with soluble leishmania antigen (SLA). SLA stimulation was initially observed to increase the expression of TLR2 and TLR4 in monocytes from CL patients compared to unstimulated cultures. Moreover, the neutralization of TLR2 and TLR4 decreased TNF expression in monocytes after stimulation with SLA. These results seem to indicate that, in our *in vivo* experimental model, even after inducing infection and the partial clearance of parasites, the presence of SLA is able to continue stimulating the immune response by increasing the expression TLR2 and TLR4, in addition to the production of inflammatory molecules (32).

Finally, as TLR2 and TLR4 have been documented in CL patient lesions (65, 66), we investigated the role of TLR2 and TLR4 receptors in lesion biopsies obtained from CL patients. Some studies have been shown the TNF and IL-1β is highly expressed in lesions from CL patients (31, 67). In this study biopsied cells were observed to express TLR2 and TLR4, while treatment with anti-TLR2 and anti-TLR4 decreased the levels of TNF, IL-1β and CXCL9 production by these cells.

The present findings provide convincing evidence that the blockade of TLR receptors can attenuate the inflammatory response observed in human cutaneous leishmaniasis caused by *L. braziliensis.* As several studies have demonstrated the use of this approach in the treatment of inflammatory diseases (68), our results present novel perspectives for the development of immunomodulatory therapies that may complement conventional treatment options for cutaneous leishmaniasis.

## Data Availability Statement

The raw data supporting the conclusions of this article will be made available by the authors, without undue reservation.

## Ethics Statement

The studies involving human participants were reviewed and approved by Institutional Review Board of the Professor Edgard Santos University Hospital Complex. The patients/participants provided their written informed consent to participate in this study.

## Author Contributions

PC and OB participated equally in the study design and in the writing of the manuscript. PC and AD participated equally in all of the experiments. WO participated in the human monocytes infection and processing of samples on the flow cytometer. LG is a physician and participated in the diagnostic of the patients in the endemic area. CB participated in the study design and in the discussion of the results. EC and OB are the principal investigators of this work and followed the work from the beginning to the end and also participated in the writing of the manuscript. All authors contributed to the article and approved the submitted version.

## Funding 

This work was supported by the National Institutes of Health (AI 136032 to EC). EC and OB are senior researchers at the Brazilian Council for Scientific and Technological Development (Conselho Nacional de Desenvolvimento Científico e Tecnológico (CNPq). 

## Conflict of Interest

The authors declare that the research was conducted in the absence of any commercial or financial relationships that could be construed as a potential conflict of interest.

## Publisher’s Note

All claims expressed in this article are solely those of the authors and do not necessarily represent those of their affiliated organizations, or those of the publisher, the editors and the reviewers. Any product that may be evaluated in this article, or claim that may be made by its manufacturer, is not guaranteed or endorsed by the publisher.

## Supplementary Material

Supplementary Figure 1 Frequency of monocytes infected with *L. braziliensis* GFP ^(+)^ after TLR2 and TLR4 neutralization. Monocytes from CL patients (n=8) were treated or not with anti-TLR2 and anti-TLR4 antibodies, then infected with *L. braziliensis* GFP ^(+)^. **(A)** Representative gating strategy using FMO (Florescence Minus One). **(B)** Frequency of GFP+ cells after neutralization of TLR2 and/or TLR4. All p values were obtained using Wilcoxon signed-rank test; **p<0.01.
