# Supplementary figures and images for: Blockade of TLR2 and TLR4 Attenuates Inflammatory Response and Parasite Load in Cutaneous Leishmaniasis
Source: Front Immunol. 2021 Oct 6;12:706510. doi: 10.3389/fimmu.2021.706510 (PMC8526941; doi:10.3389/fimmu.2021.706510)

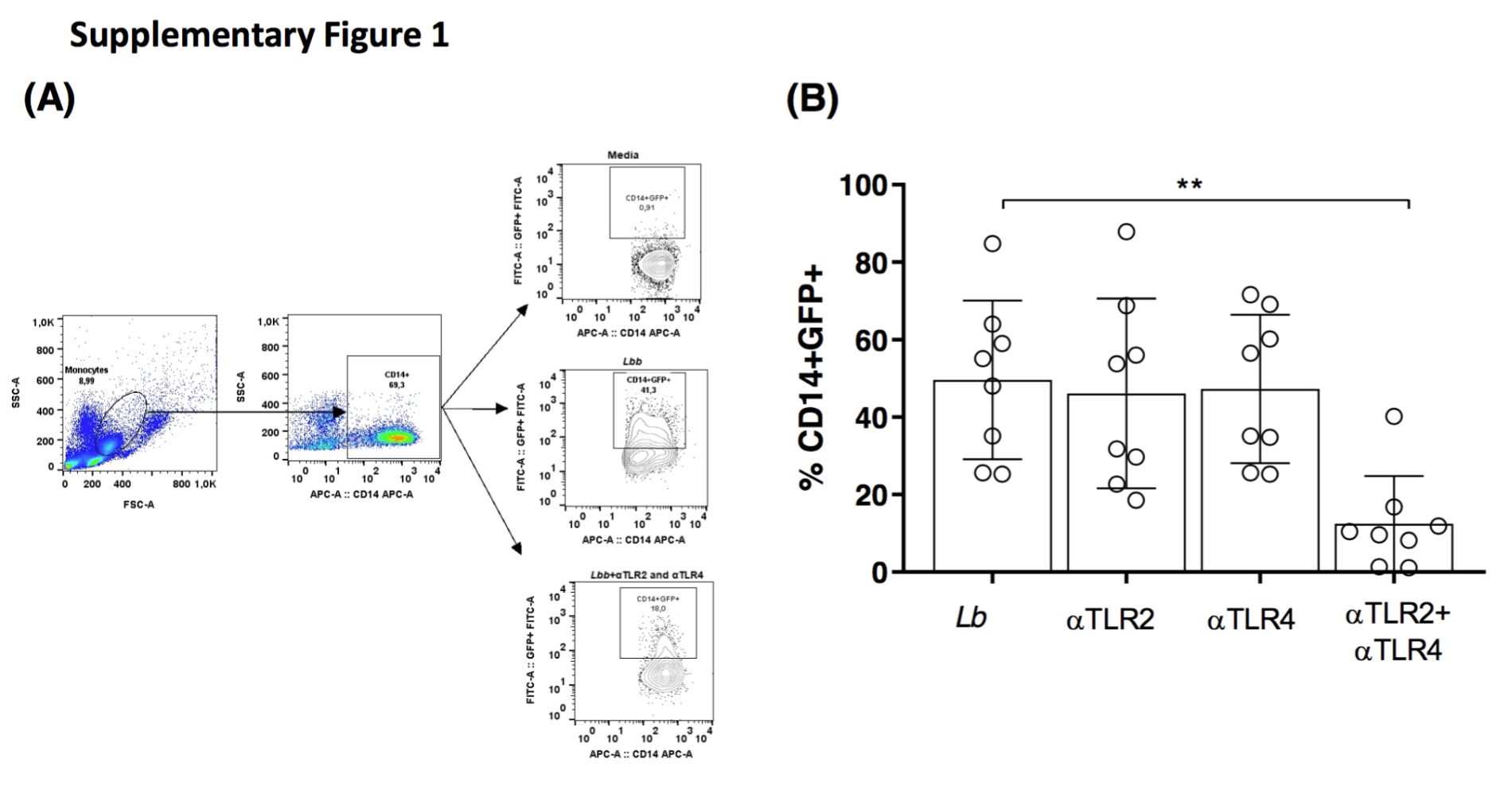

Supplement: Supplementary Figure 1 — Frequency of monocytes infected with L. braziliensis GFP (+) after TLR2 and TLR4 neutralization. Monocytes from CL patients (n=8) were treated or not with anti-TLR2 and anti-TLR4 antibodies, then infected with L. braziliensis GFP (+). (A) Representative gating strategy using FMO (Florescence Minus One). (B) Frequency of GFP+ cells after neutralization of TLR2 and/or TLR4. All p values were obtained using Wilcoxon signed-rank test; **p<0.01. [file Image_1.jpeg]
